# Boosting the Catalytic Performance of AuAg Alloyed Nanoparticles Grafted on MoS_2_ Nanoflowers through NIR-Induced Light-to-Thermal Energy Conversion

**DOI:** 10.3390/nano13061074

**Published:** 2023-03-16

**Authors:** Sara Rodríguez-da-Silva, Abdel Ghafour El-Hachimi, José M. López-de-Luzuriaga, María Rodríguez-Castillo, Miguel Monge

**Affiliations:** Department of Chemistry, Centro de Investigación en Síntesis Químicas (CISQ), University of La Rioja, C/Madre de Dios 53, E-26006 Logroño, La Rioja, Spain

**Keywords:** gold, silver, molybdenum disulfide, photothermal effect, catalysis

## Abstract

MoS_2_ nanoflowers (NFs) obtained through a hydrothermal approach were used as the substrate for the deposition of tiny spherical bimetallic AuAg or monometallic Au nanoparticles (NPs), leading to novel photothermal-assisted catalysts with different hybrid nanostructures and showing improved catalytic performance under NIR laser irradiation. The catalytic reduction of pollutant 4-nitrophenol (4-NF) to the valuable product 4-aminophenol (4-AF) was evaluated. The hydrothermal synthesis of MoS_2_ NFs provides a material with a broad absorption in the Vis-NIR region of the electromagnetic spectrum. The in situ grafting of alloyed AuAg and Au NPs of very small size (2.0–2.5 nm) was possible through the decomposition of organometallic complexes [Au_2_Ag_2_(C_6_F_5_)_4_(OEt_2_)_2_]_n_ and [Au(C_6_F_5_)(tht)] (tht = tetrahydrothiophene) using triisopropilsilane as reducing agent, leading to nanohybrids **1**–**4**. The new nanohybrid materials display photothermal properties arising from NIR light absorption of the MoS_2_ NFs component. The AuAg-MoS_2_ nanohybrid **2** showed excellent photothermal-assisted catalytic activity for the reduction of 4-NF, which is better than that of the monometallic Au-MoS_2_ nanohybrid **4**. The obtained nanohybrids were characterised by transmission electron microscopy (TEM), High Angle Annular Dark Field—Scanning Transmission Electron Microscopy—Energy Dispersive X-ray Spectroscopy (HAADF-STEM-EDS), X-ray photoelectron spectroscopy and UV-Vis-NIR spectroscopy.

## 1. Introduction

The development of new nanostructures displaying optimal light-to-thermal energy conversion properties is gaining increasing attention in different fields including environmental applications such as photothermal elimination of persistent wastewater pollutants [1], H_2_ generation [2] and water desalination [3], or biomedical applications for the elimination of antibiotic-resistant microorganisms [4] and cancer treatment through photothermal therapy [5], among others.

A promising research line consists of the fabrication of hybrid multifunctional nanostructures displaying very efficient UV, Visible and/or NIR light harvesting capacity combined with high catalytic performance. In this sense, by using the generated heat in the photothermal conversion by one of the components of a given nanohybrid, the catalytic performance of a second component could be strongly boosted [6]. Therefore, the purposeful combination of such components in one nanomaterial would give rise to photothermal-assisted catalysts displaying improved properties. In this context, the elimination of water pollutants such as nitroaromatic compounds is a major challenge because of two reasons, namely: (i) the high toxicity of these molecules in water, and (ii) the wide range of applications of the products of catalytic reduction (aminoaromatics) in different fields as inhibitors for corrosion, drying agents or as drug precursors [7]. Therefore, the improved catalysed reduction of 4-nitrophenol (4-NP) to 4-aminophenol (4-AP) by photothermal-assisted hybrid catalysts constitute a very interesting challenge.

Among nanomaterials displaying efficient light-to-thermal energy conversion ability, 2D-transition metal dichalcogenides, such as MoS_2_, display excellent potential [8,9,10,11]. These materials can be used in the design of photothermal-assisted catalysts. Indeed, in recent work by Lu et al. [12] the authors show how photothermically heated porous MoS_2_ nanoparticles by NIR laser irradiation accelerates the catalytic reduction of 4-NP to 4-AP concerning non-irradiated MoS_2_ NPs [13], displaying the MoS_2_ NPs themselves the photothermal and catalytic property at the same time.

On the other hand, noble metal NPs display very good performance as catalysts in the reduction of 4-NP to 4-AP. Among them, Au NPs have been the metal of choice in many cases [14] compared to others such as Ag [15], Pd [16], Pt [17] or Ni [18] NPs because the activity of gold in this transformation only takes place in the nanometre size scale and because Au NPs are active under very mild conditions. However, in the last years, the use of bimetallic NPs has shown that enhanced catalytic performance can be achieved compared to their monometallic counterparts [19]. Thus, the formation of alloyed AuAg NPs stabilized by different substrates like carbon spheres [20], starch [21] or silica coating [22] gives rise to catalysts displaying an improved performance when compared to the monometallic ones. The factors affecting this improved performance are thought to be synergistic electronic and structural effects. In this regard, Menezes et al. [19] reported on a comparative study in which the catalytic activity of AuAg NPs in the 4-NP reduction reaction was better than the ones obtained for pure Au and pure Ag NPs. A very important point is that comparable nanoparticles in terms of size and surface environment were needed. The higher activity of AuAg NPs was ascribed to an electron enrichment in the gold near the interface by neighbouring silver atoms, which favours the reduction of the reactant on the gold sites.

There have been some reports regarding the fabrication and use of catalysts/photocatalysts based on monometallic Au or Ag-MoS_2_ hybrid nanostructures. For example, Au NPs deposited on the surface of MoS_2_ nanostructures have been tested as catalysts (without photothermal heating), in the reduction of 4-NP to 4-AP [23], and in the plasmon-enhanced photoelectrochemical or photocatalytic water-splitting reaction [24]. On the other hand, Ag NPs-MoS_2_ nanostructures have been employed as photocatalysts for visible-light-driven H_2_ evolution [25] or methylene blue dye degradation [26]. However, as far as we are aware, there is no study reported to date based on the combination of alloyed AuAg NPs grafted on the surface of MoS_2_ 2D nanostructures. In this regard, we have developed an organometallic approach for the synthesis of size and shape-controlled Au and AuAg NPs of different sizes and shapes by the reduction of pentafluorophenyl precursors [Au(C_6_F_5_)(tht)] (tht = tetrahydrothiophene) [27] and [Au_2_Ag_2_(C_6_F_5_)_4_(OEt_2_)_2_]_n_ [28], respectively. The formation of Au and AuAg NPs from these precursors has also allowed the synthesis of very efficient plasmonic photocatalysts for the depletion of persistent drugs in wastewater, through the formation of the metal NPs at the surface of semiconductor carbon nitride 2D nanosheets [29,30], or for photocatalytic dehydrogenation of NH_3_·BH_3_ and H_2_ release using wavy AuAg nanorods [31]. Following this idea and given the excellent photothermal properties of MoS_2_, we wondered whether the inclusion of bimetallic AuAg NPs of small size, which are superior catalysts in the transformation of 4-NP to 4-AP, would produce a new type of hybrid material in which a synergistic effect between the photothermal heating produced at MoS_2_ NFs would increase, even more, the efficiency of the grafted AuAg NPs in the catalytic reduction of 4-NP.

Therefore, we report herein the synthesis of novel AuAg-MoS_2_ NFs (**1**, **2**) hybrid materials by the reduction of the organometallic complex [Au_2_Ag_2_(C_6_F_5_)_4_(OEt_2_)_2_]_n_ in the presence of triisopropylsilane as reducing agent. We have also synthesized the analogous monometallic Au-MoS_2_ NFs hybrid materials (**3**, **4**) by reducing complex [Au(C_6_F_5_)(tht)] in similar conditions, to prove the better catalytic performance of alloyed AuAg NPs compared to the monometallic Au ones. We report on the photothermal and catalytic properties of these new hybrid materials towards the reduction of 4-NP to 4-AP.

## 2. Materials and Methods

### 2.1. Materials and Reagents

Thiourea (98%) was provided by Panreac (Barcelona, Spain). Ammonium molybdate tetrahydrate (99.5%) was purchased from Glentham (Corsham, UK) and poly (ethylene glycol) from Frontier Specialty Chemicals (Beijing, China). Tethahydrofuran and triisopropilsilane (98%), used in the synthesis of nanoparticles, were provided by VWR Chemicals and Sigma Aldrich, respectively. All chemicals were used as received and no further purification was needed. The precursors [Au_2_Ag_2_(C_6_F_5_)_4_(OEt_2_)_2_]_n_ and [Au(C_6_F_5_)(tht)] (tht = tetrahydrothiophene) used for the synthesis of metallic NPs were synthesized by standard procedures reported previously [32,33].

### 2.2. Synthesis of Nanohybrid Materials

#### 2.2.1. Synthesis of MoS_2_ NFs

MoS_2_ NFs were synthesized according to a previously reported methodology [34]. Briefly, 0.1766 g of (NH_4_)_6_Mo_7_O_24_·4H_2_O and 0.5 g of polyethyleneglycol (PEG, 40,000 MW) were dissolved in 20 mL of H_2_O (milli-Q) leading to a clear colourless solution. Then, a 10 mL solution containing 2 mmol of thiourea, SC(NH_2_)_2_, was added, leading to a homogeneous solution. The mixture was transferred to a stainless-steel autoclave with a closed Teflon container, and it was placed in an oven preheated to 180 °C for 12 h. Finally, a black solution was obtained, which was centrifuged (10 min, 5000 rpm) and washed twice with an EtOH/H_2_O solution (50% *v*/*v*). Evaporation to dryness led to the formation of MoS_2_ NFs as a black solid.

#### 2.2.2. Synthesis of AuAg-MoS_2_ Nanohybrid Materials (**1**, **2**)

To a suspension of MoS_2_ NFs (15 mg) in 15 mL of tetrahydrofuran (THF), 1 mL of a THF solution of [Au_2_Ag_2_(C_6_F_5_)_4_(OEt_2_)_2_]_n_ (leading to 5% wt of Au and Ag metals concerning MoS_2_) was added and mixed through stirring for 5 min at room temperature. Then, 10 μL of triisopropylsilane (TIPS) was added to the reaction mixture and the suspension was stirred and heated under reflux at 66 °C, in dark conditions, for 1 h. After that, the formed solid suspension of nanohybrid **1** was centrifuged and washed with THF twice, leading to a black solid by evaporation of the remaining solvent.

The same procedure was repeated to obtain AuAg-MoS_2_ nanohybrid **2** but adding in this case 2 μL of a THF solution of [Au_2_Ag_2_(C_6_F_5_)_4_(OEt_2_)_2_]_n_ (leading to 1% wt of Au and Ag metals concerning MoS_2_).

#### 2.2.3. Synthesis of Au-MoS_2_ Nanohybrid Materials (**3**, **4**)

To a suspension of MoS_2_ NFs (15 mg) in 15 mL of tetrahydrofuran (THF), 1 mL of a THF solution of [Au(C_6_F_5_)(tht)] (tht = tetrahydrothiophene) (leading to 5% wt of Au metal concerning MoS_2_) was added and mixed through stirring for 5 min at room temperature. Then, 10 μL of triisopropylsilane (TIPS) was added to the reaction mixture and the suspension was stirred and heated under reflux at 66 °C, in dark conditions, for 1 h. After that, the formed solid suspension of nanohybrid **3** was centrifuged and washed with THF twice, leading to a black solid by evaporation of the remaining solvent.

The same procedure was repeated to obtain Au-MoS_2_ nanohybrid **4** but adding in this case 2 μL of a THF solution of [Au(C_6_F_5_)(tht)] (leading to 1% wt of Au metal concerning MoS_2_).

### 2.3. Characterization of Nanohybrids

Absorption UV-Vis-NIR spectra of water dispersions of all the obtained nanohybrid materials were recorded on a Shimadzu UV-3600 spectrophotometer. Transmission Electron Microscopy (TEM) samples were drop-casted from ethanol dispersions (2–3 drops) to carbon-coated Cu grids. The corresponding TEM micrographs were acquired with a Tecnai T20 (ThermoFisher Scientific) at a working voltage of 200 kV. In addition, High Angle Annular Dark Field—Scanning Transmission Electron Microscopy (HAADF-STEM) images were registered with an Analytical Titan (FEI) at a working voltage of 300 kV, coupled with a HAADF detector (Fischione). Using this mode of measurement, the signal intensity is proportional to Z^2^. Therefore, heavy elements like gold or silver display a brighter contrast compared to lighter elements. In addition, to analyze the composition of the materials, we registered X-ray Energy Dispersive Spectra (EDS) using an EDAX detector or with an Ultim Max detector (Oxford). XPS experiments were performed with a Kratos AXIS Supra spectrometer, using a monochromatized Al Kα source (1486.6 eV) operating at 12 kV and 10 mA. Survey spectra were registered at an analyzer pass energy of 160 eV, while narrow scans were acquired at constant pass energy of 20 eV and steps of 0.1 eV. The photoelectrons were detected at a take-off angle F = 0° concerning the surface normal. Basal pressure was below 5 × 10^−9^ Torr. The spectra were obtained at room temperature. The binding energy (BE) scale was internally referenced to the C 1s peak (BE for C–C = 284.8 eV). The data treatment was carried out with the Casa XPS software using the specific relative sensitivity factor library that the software has for Kratos equipment. To calculate the atomic concentrations a Shirley-type background subtraction was used. The 4f region was used for Au; the 3d region was used for Mo and Ag and the 2p region was used for S.

### 2.4. Computational Study

All DFT results were obtained using the PWscf code of the Quantum ESPRESSO package [35], which uses the projector-augmented wave (PAW) pseudopotential [36], within Perdew-Burke-Ernzerhof (PBE) functional [37,38] of generalized gradient approximation (GGA) level of theory. The integration of the Brilloune zone was sampled by the automatically generated 4 × 4 × 1 Monkhorst-Pack K-point grids. The convergence threshold of total energy and Hellmann–Feynman force was chosen to be 10^−8^ Ry, and 10^−3^ Ry/bohr, respectively. The maximum energy and density of charge cut offs for the plane wave expansion is set to 50 Ry and 1000 Ry, respectively.

The change of electronic density (Δρ) was computed according to the following expression:

Δρ(r) = ρ(r)adsorbate/MoS_2_ − ρ(r)adsorbate − ρ(r)MoS_2_

where ρ(r)adsorbate/MoS_2_, ρ(r)adsorbate and ρ(r)MoS_2_ are charge densities of adsorbate (metallic clusters) interaction onto MoS_2_ monolayer, cluster metallic isolated, and MoS_2_ surfaces modelled, respectively.

The MoS_2_ 1T phase is the predominant one. It was chosen to highlight our experimental observation. The unit cell of the MoS_2_-1T crystalizes in a P3-m1 symmetry, and in our calculation, we used the optimized lattice parameters a = b = 3.25 Å. Moreover, to avoid spurious interaction between images in the monolayer, we choose a separation distance of 20 Å, along the z direction. The unit cell of MoS_2_ is extended to 4 × 4 × 1, which contains 16 Mo atoms and 32 S atoms, in this (4 × 4) supercell. We adsorbed two different subnanoclusteres: Au_2_Ag and Au_3_.

The charge transfer analysis of the Au_2_Ag-MoS_2_ and Au_3_-MoS_2_ nanohybrids was performed using a Bader analysis of the electron densities [39,40].

### 2.5. Photothermal Effect Measurement

The photothermal effect was measured using an MDL-III-808R NIR laser working with an 808 nm wavelength and 2.5 W·cm^−2^ irradiation power. 1 mL of a solution of MoS_2_ NFs or the nanohybrid materials **1**–**4** (0.2 mg·mL^−1^) were placed in a quartz cuvette and it was irradiated for 10 min. Temperature changes were monitored using a FLIR E6-XT thermographic camera.

### 2.6. Photothermal-Assisted Catalytic Activity Measurement

The catalytic reduction of 4-NP to 4-AP in the presence of an excess of NaBH_4_ and using pristine MoS_2_ NFs and nanohybrids **2** or **4**, was carried out in the dark or under 808 nm NIR light laser irradiation. For this, a 4-NP solution (100 μL, 2.5 mM) was added to 2.5 mL of miliQ water in a quartz cuvette. Then, 20 μL of a catalyst solution of 4.5 mg·mL^−1^ concentration was added to the cuvette. The mixture was stirred, and laser irradiated for 15 min until a constant temperature was reached. A freshly prepared NaBH_4_ solution (150 μL, 1 M) was quickly injected into the reaction mixture. The UV spectrum was monitored until all 4-NP was converted into 4-AP.

## 3. Results and Discussion

### 3.1. Synthesis and Characterisation of Nanohybrids

**Scheme 1:**
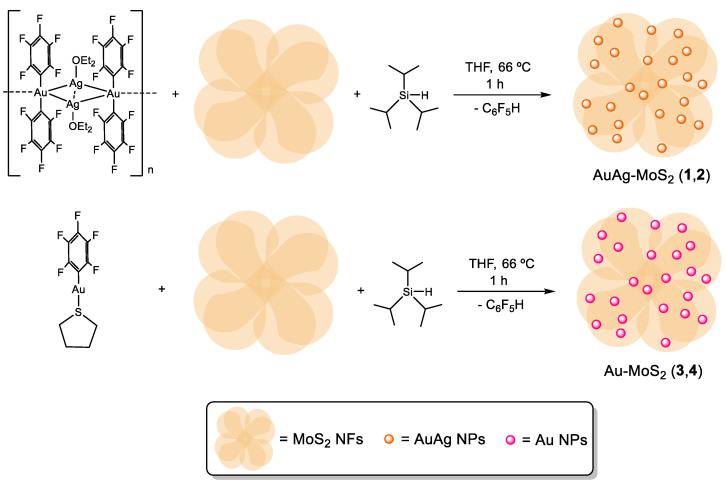
Synthesis of AuAg-MoS_2_ nanohybrids **1** and **2** by reduction of complex [Au_2_Ag_2_(C_6_F_5_)_4_(OEt_2_)_2_]_n_ and Au-MoS_2_ nanohybrids **3** and **4** by reduction of complex [Au (C_6_F_5_)(tht)] in tetrahydrofuran (THF) at 66 °C, using triisopropylsilane as reducing agent.

The need for facile methods for the synthesis of well-controlled nanohybrid materials prompted us to test the reduction of organometallic complexes [Au_2_Ag_2_(C_6_F_5_)_4_(OEt_2_)_2_]_n_ and [Au(C_6_F_5_)(tht)] using triisopropylsilane as reducing agent in the presence of MoS_2_ NFs, previously prepared through a hydrothermal route. Scheme 1 depicts the synthetic approach.

In the first step, MoS_2_ NFs were prepared through a hydrothermal approach, as previously reported (vide supra). In a second step, we carried out the decomposition of the organometallic precursor [Au_2_Ag_2_(C_6_F_5_)_4_(OEt_2_)_2_]_n_ (5% (**1**) or 1% (**2**) of Au-Ag weight content added to MoS_2_ NFs), under 1h of refluxing reaction conditions in tetrahydrofuran and with the addition of an excess of triisopropilsilane reducing agent, leading to AuAg-MoS_2_ nanohybrids **1** and **2**. For the sake of comparison, we also carried out the same strategy for the synthesis of Au-MoS_2_ nanohybrids **3** and **4**, through the reduction of the organometallic complex [Au(C_6_F_5_)(tht)] (5% (**3**) or 1% (**4**) of Au-Ag weight content added to MoS_2_ NFs).

**Figure 1:**
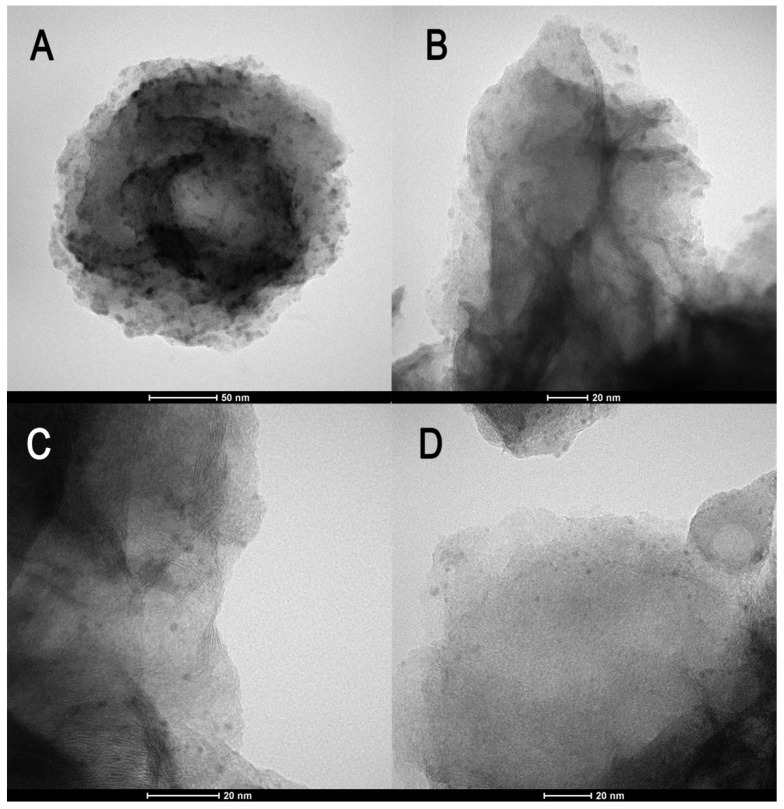
TEM images of AuAg-MoS_2_ nanohybrid **1** (**A**,**B**) and **2** (**C**,**D**).

The isolated nanohybrid materials were characterized through TEM and HAADF-STEM. Figure 1 displays TEM images of the formed AuAg-MoS_2_ nanohybrids **1** and **2**. The low magnification images show the homogeneous distribution of spherical NPs of 2.5 ± 0.9 nm size for **1** and 2.1 ± 0.3 nm size for **2** grafted on the surface of MoS_2_ NFs (see size histograms in Supplementary Materials, Figure S1).

**Figure 2:**
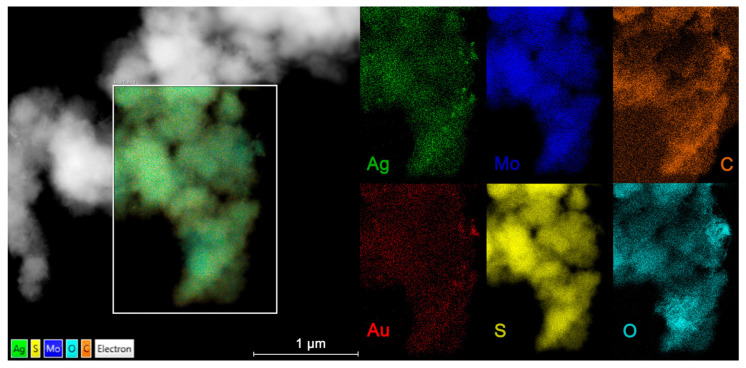
HAADF-STEM-EDS analysis of AuAg-MoS_2_ nanohybrid **1** for all elements (left) and for individual elements O (cyan), C (orange), S (yellow), Mo (blue), Ag (green) and Au (red).

The HAADF-STEM images of AuAg-MoS_2_ nanohybrid **1** (see Figure S2 in Supplementary Materials) show that the overlapping layers that characterized the starting MoS_2_ NFs are kept separated by a distance of ca. 1.00 nm. Therefore, an expansion between adjacent layers due to the presence of PEG polymer is maintained unchanged with the introduction of the bimetallic AuAg NPs. In addition, the atomic planes of close fcc packing centered on gold and silver can be observed. We performed a HAADF-STEM-EDS analysis of AuAg-MoS_2_ nanohybrid **1** to confirm the composition of the nanohybrid. Figure 2 shows the EDS spatial elemental composition for elements C, O, Mo, S, Au, and Ag individually, showing that all elements are distributed homogeneously within the sample. The composed images of all elements mapped in the same image are also shown. The EDS weight % composition analysis shows a 1.3% wt of Ag and 0.7% wt of Au.

**Figure 3:**
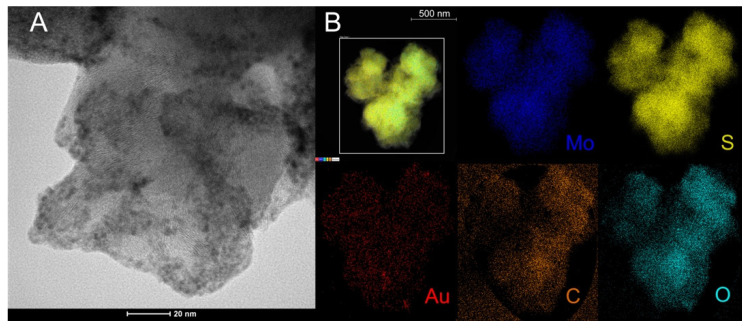
TEM images of Au-MoS_2_ nanohybrid **3** (**A**) and HAADF-STEM-EDS analysis of Au-MoS_2_ nanohybrid **3** (**B**) and for individual elements O (cyan), C (orange), S (yellow), Mo (blue), and Au (red).

The TEM images of Au-MoS_2_ nanohybrid **3** are shown in Figure 3 (see Supplementary Materials for TEM images of Au-MoS_2_ nanohybrid **4** and size histograms in Figure S3). The TEM images show the homogeneous distribution of very small size spherical NPs of 2.7 ± 0.7 nm size for **3** and 2.1 ± 0.9 nm size for **4**. It is worth mentioning that the synthetic approach provides AuAg and Au NPs of almost the same size, which makes this approach interesting for further comparing the catalytic composition-dependent properties of alloyed bimetallic or monometallic NPs of similar size (vide infra). Again, the analysis of HAADF-STEM-EDS images obtained for Au-MoS_2_ nanohybrid **3** confirms the spatial composition of the nanohybrid. The EDS spatial elemental composition shown in Figure 3 for isolated elements C, O, Mo, S and Au, displays again a homogeneous distribution. The composed images of all elements mapped in the same image confirm this trend.

**Figure 4:**
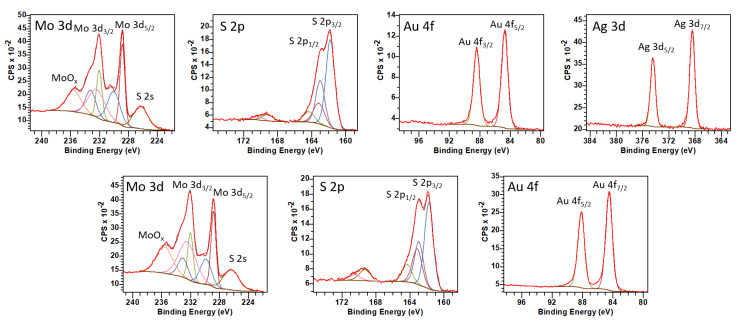
Narrow XPS spectra for Mo 3d, S 2p, Au 4f and Ag 3d for AuAg-MoS_2_ nanohybrid **1** (**top**) and Mo 3d, S 2p and Au 4f for Au-MoS_2_ nanohybrid **3** (**bottom**).

The detailed information on the surface elemental composition and the chemical states of the nanohybrids AuAg-MoS_2_ **1** and Au-MoS_2_ **3** was described through X-ray Photoelectron Spectroscopy (XPS). We have included the survey spectra for MoS_2_ NFs and nanohybrids **1** and **3**; their % atomic composition and the high-resolution spectra of Mo 3d and S 2p for MoS_2_ NFs in the Supplementary Materials (see Figures S4–S7 and Table S1). The atomic % composition analysis shows a 1.3% atomic Ag and 0.7% atomic Au composition, whereas a 2.07 atomic % is observed for Au in nanohybrid **3**. The survey spectra for nanohybrids **1** and **3** display the characteristic peaks of MoS_2_, PEG polymer and low intensity peaks corresponding to Au and Ag metals (or only Au in the case of **3**). Figure 4 depicts the high-resolution spectra for **1** and **3**, including elements Mo 3d, S 2p, Au 4f and Ag 3d (only in the case of **1**).

The narrow XPS spectra for the Mo 3d region obtained for nanohybrids **1** and **3** (Figure 4) display two intense peaks (red and green lines) at 228.8 and 232.0 eV for both and at 229.0 and 232.2 eV for pristine MoS_2_ NFs (Figure S7). These peaks are assigned to Mo 3d_5/2_ and Mo 3d_3/2_ binding energies of Mo^4+^ in octahedral metallic 1T-MoS_2_ phase separated by ca. 3.2 eV. In addition, the deconvolution of the signals is in agreement with the presence of two peaks (cyan and blue lines) slightly shifted to higher energies (230.0 and 233.2 eV for **1**, 229.9 and 233.1 eV for **3** and 230.2 and 233.4 eV for MoS_2_ NFs) that correspond to Mo 3d_5/2_ and Mo 3d_3/2_ binding energies of Mo^4+^ in (separated by 3.2 eV) in hexagonal 2H-MoS_2_ phase, which displays semiconducting properties. The higher energy peaks (pink and orange lines) at 232.4 and 235.6 eV for **1**, 232.5 and 235.7 eV for **3** and 232.6 and 235.8 eV for MoS_2_ NFs are related to the minor formation of MoO_x_ species in a +6-oxidation state and assigned to Mo 3d_5/2_ and Mo 3d_3/2_ binding energies (separated by 3.2 eV). Also, both the branching ratio of 6:4 for Mo 3d_5/2_ and Mo 3d_3_ signals and comparable FWHM values support this assignment. The analysis of the Mo 3d peaks agrees with a mixture of 1T metallic and 2H semiconducting MoS_2_ phases composition in the precursor MoS_2_ NFs and in samples **1** and **3**. On the other hand, we have analysed the S 2p region for **1** and **3** and MoS_2_ NFs. In this case, we observe peaks related to the 1T-MoS_2_ phase (cyan and blue lines) at 161.8 and 163.0 eV for **1** and **3** and at 161.9 and 163.1 eV for MoS_2_ NFs. These peaks are assigned to S 2p_3/2_ and 2p_1/2_ binding energies in octahedral metallic 1T-MoS_2_ phase separated by ca. 1.2 eV The deconvolution of the peaks also shows the signals (read and green lines) corresponding to S 2p_3/2_ and 2p_1/2_ binding energies in 2H-MoS_2_ phase at 163.1 and 164.3 eV for **1** and **3**, and at 163.5 and 164.7 eV for MoS_2_ NFs (separated ca. 1.2 eV), in agreement with the 1T and 2H phases’ mixture [41]. Also, low intensity peaks (green and pink lines) at 169.2 and 170.4 (**1**), 169.3 and 170.5 (**2**) and 168.7 and 169.9 for MoS_2_ NFs can be attributed to S 2p_3/2_ and 2p_1/2_ binding energies of S^6+^ (probably sulfate) species. The deconvolution of the Au 4f spin-orbit doublets for **1** and **3** is also shown in Figure 4. The narrow experimental profiles can be fitted to one intense spin-orbit doublet (red and green lines, separated ca. 3.7 eV) at 84.7 and 88.4 for **1** or 84.5 and 88.2 eV for **3**. The slightly higher Au 4f_7/2_ binding energy detected in the high-resolution spectra for nanohybrids **1** and **3** at 84.7 and 84.5 eV (pure gold is 84.0 eV), respectively, would agree with a charge transfer between Au and S, proving the formation of Au-S bonds [42]. In the narrow spectrum for the Ag 3d region for **1** a unique spin-orbit doublet separated 6.0 eV appears at 368.4 and 374.4 eV (red and green lines), which can be assigned to metallic silver [43].

The Raman spectra of MoS_2_ NFs and nanohybrids **1**–**4** confirm the co-existence of 1T and 2H phases of MoS_2_ (see Supplementary Materials, Figure S8). The peaks related to 2H-MoS_2_ appear at ca. 374, 406 and 460 cm^−1^ and correspond to E^1^_2g_ (S-Mo-S in-plane vibrations), A_1g_ (out-of-plane vibrations of S atoms and a longitudinal acoustic mode), respectively. The profile related to the 1T phase of MoS_2_ appears at ca. 147, 192 and 330 cm^−1^, corresponding to phonon modes: at 210, 235 cm^−1^, corresponding to longitudinal acoustic modes, and at 280, and 405 cm^−1^, corresponding to E_1g_ and A_1g_ vibrations, respectively [9].

**Figure 5:**
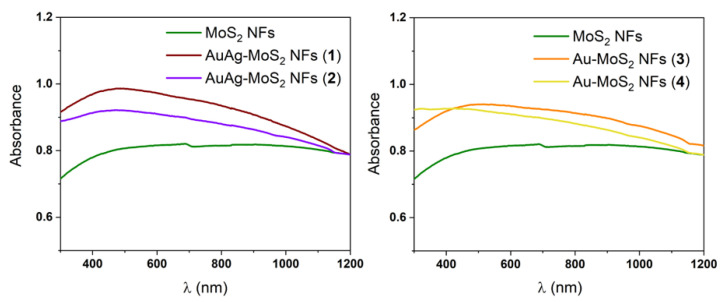
Solid UV-Vis spectra for pristine MoS_2_ NFs and nanohybrids **1**–**2** (**left**) and **3**–**4** (**right**).

The UV-Vis-NIR spectra for AuAg-MoS_2_ nanohybrids **1**, **2** and Au-MoS_2_ nanohybrids **3**, **4** show that all the materials absorb strongly in the whole UV-Vis-NIR range. The presence of AuAg or Au spherical NPs on the MoS_2_ NFs produces an increase of absorbance in the visible range (between 350 and 1200 nm) compared to the pristine MoS_2_ nanoflowers, as shown in Figure 5. As previously reported, a monotonic change would agree with the presence of a pure 1T metallic phase [44]. The presence of broad shoulders and non-monotonic profiles also agrees with the above-mentioned mixture of 1T and 2H phases. The plasmonic absorptions arising from spherical AuAg NPs (around 470 nm in solution for a 50:50 Au: Ag composition) and spherical Au NPs (around 530 nm) would be included in the increased absorbance regions observed for nanohybrids **1**–**4**.

### 3.2. Photothermal Properties

**Figure 6:**
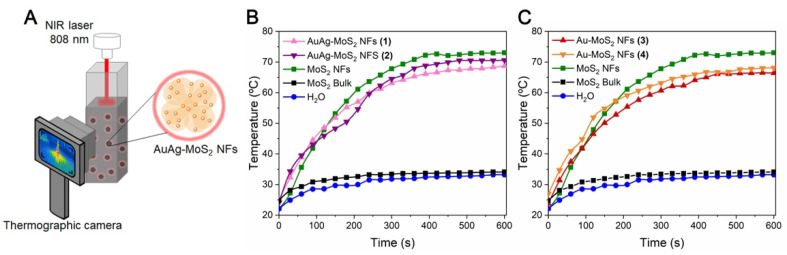
Set-up for the measurement of the photothermal conversion of NIR laser light (808 nm, 2.5 W·cm^−2^) into thermal energy (**A**); photothermal heating curves for pure water solvent, bulk MoS_2_, MoS_2_ NFs and AuAg-MoS_2_ NFs **1** and **2** (**B**) or Au-MoS_2_ NFs **3** and **4** (**C**).

After the characterization of MoS_2_ NFs and nanohybrids **1**–**4**, we studied their photothermal properties and compared them with that of bulk MoS_2_. Figure 6A depicts the setup used for the measurement of the light-to-energy conversion using a NIR laser light source (808 nm, 2.5 W·cm^−2^) and the monitoring of the temperature increase using a thermographic camera. The photothermal conversion performance of AuAg-MoS_2_ nanohybrids **1** and **2** depicted in Figure 6B displays the photothermal heating curves with a maximum value after 600 s slightly lower than that of MoS_2_ NFs (73 °C), reaching 69 and 70 °C for **1** and **2**, respectively. Similar behaviour is observed for Au-MoS_2_ NFs **3** and **4**, with temperatures of 66 and 68 °C, respectively. The photothermal heating curve of bulk MoS_2_ shows an almost negligible photothermal conversion (34 °C) under the same conditions (black curve), being only slightly higher than that of pure water (33 °C). We checked the stability of the nanohybrids **3** and **4** by collecting successive on-off irradiation-cooling cycles (see Supplementary Materials, Figure S9). The obtained curves show that the maximum temperatures reached after the cooling of the samples were kept, suggesting good stability and recyclability of these materials when used, for instance, for catalytic applications.

### 3.3. Photothermal-Assisted Catalytic Activity

This catalytic study aims to check the performance of modified MoS_2_ NFs catalyst in a benchmark reaction such as the catalytic reduction of 4-nitrophenol (4-NP) into 4-aminophenol (4-AP) when catalytic noble metal AuAg or Au NPs are incorporated to this 2D nanostructures. Considering the interesting photothermal properties displayed by these nanohybrids a major question was to prove the positive influence of the local photothermal heating provided by MoS_2_ NFs for boosting the efficiency of this catalytic transformation.

**Figure 7:**
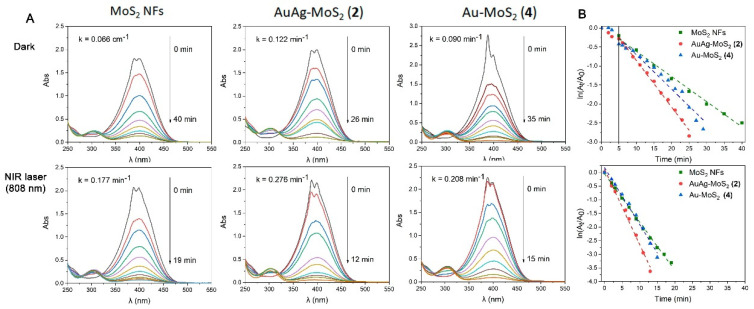
Time-monitored absorption spectra for the reduction of 4-NP to 4-AP in different conditions (darkness or irradiation with an 808 nm NIR laser, using MoS_2_ NFs and nanohybrids **2** and **4** (**A**); Linear fit representations of ln(A_t_/A_0_) vs. time (**B**).

Therefore, we studied the catalytic reduction of 4-NP into 4-AP by NaBH_4_, in the presence of MoS_2_ NFs, AuAg-MoS_2_ nanohybrid **2** and Au-MoS_2_ nanohybrid **4**, being the catalysts the ones with less metal content among the ones reported in this work. We analysed the efficiency of these catalysts both in dark conditions and under NIR laser light irradiation of 808 nm and 2.5 W·cm^−2^ power. To evaluate the rate of conversion of 4-NP into 4-NP we monitored the catalytic reduction of 4-NP through UV-Vis absorption spectroscopy, which allows following the absorbance decrease of the band attributed to the nitrophenolate ion at ca. 400 nm and the concomitant increase of the absorption assigned to 4-AP at ca. 318 nm (Figure 7A). The representation of ln(A_t_/A_0_) vs. time (Figure 7B) provides the estimation of the rate constant (k) for each process (included in each UV-Vis monitored graphical representation), in agreement with a pseudo-first-order reduction of 4-NP into 4-AP, in the presence of an excess of NaBH_4_.

The results provide interesting insights into the catalytic performance of the reported noble metal NPs-MoS_2_ NFs hybrids. The first observation is that MoS_2_ NFs catalyze the reduction of 4-NP into 4-AP with a fitted rate constant of 0.066 min^−1^ (R^2^ = 0.99232) dark conditions and a complete transformation to 4-AP in 40 min of catalytic reduction. The same conditions but changing the catalysts to nanohybrids AuAg-MoS_2_ (**2**) and Au-MoS_2_ (**4**) produce a clear increase in the reduction rate, leading to fitted 0.122 min^−1^ (R^2^ = 0.9944) and 0.090 min^−1^ (R^2^ = 0.96008) rate constants and complete reduction times of 26 and 35 min, respectively. These results indicate that the presence of alloy AuAg NPs boosts the reduction of 4-AP, even more than Au NPs. In addition, when the catalytic processes are carried out in the presence of NIR light laser irradiation a very important increase in the reaction rates that arises from the photothermal heating of MoS_2_ NFs can be observed for all the catalysts. Thus, the fitted rate constants for MoS_2_ NFs and nanohybrids **2** and **4** are 0.177 (R^2^ = 0.99707), 0.276 (R^2^ = 0.99124) and 0.208 min^−1^ (R^2^ = 0.98645), with complete reduction times of 19, 12 and 15 min, respectively, showing the positive effect provided by the light-to-local heating transformation of the MoS_2_ NFs to the efficiency of the catalysts in addition to that arising from the metal nanoparticles.

**Figure 8:**
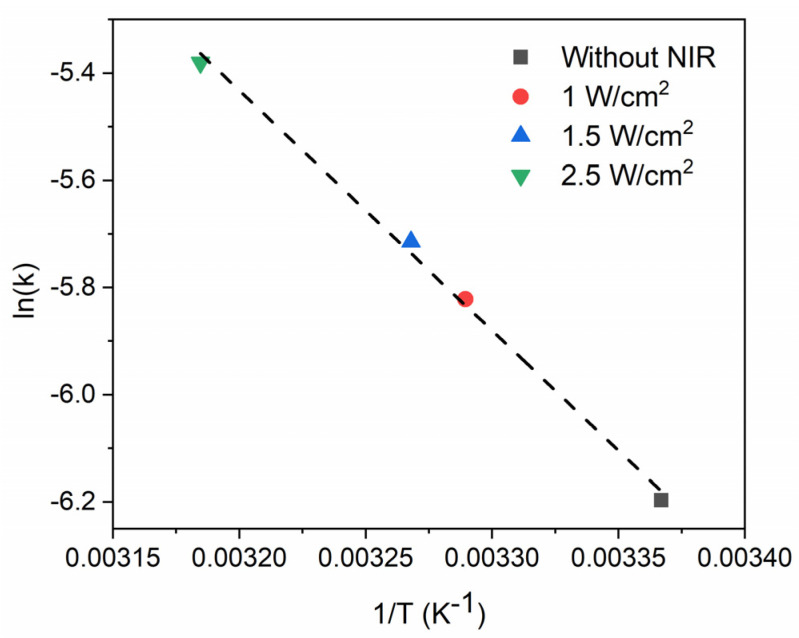
Representation of ln(k) vs. 1/T. The rate constants were obtained at different temperatures in experiments carried out under NIR irradiation with different irradiation power and in the absence of light.

We also carried out the reduction of 4-NP at different temperatures by modulating the NIR irradiation power. We calculated the rate constants k at 297, 304; 306 and 314 K (see Supplementary Materials, Figure S10) and we represented ln(k) versus 1/T (Figure 8). The slope of the linear fit of the representation provides the activation energy (E_a_) following the Arrhenius equation. The obtained E_a_ was 32.3 ± 1.3 kJ·mol^−1^ is comparable to that previously reported for porous MoS_2_ nanoparticles of 34.7 ± 3.6 kJ·mol^−1^ [12].

**Figure 9:**
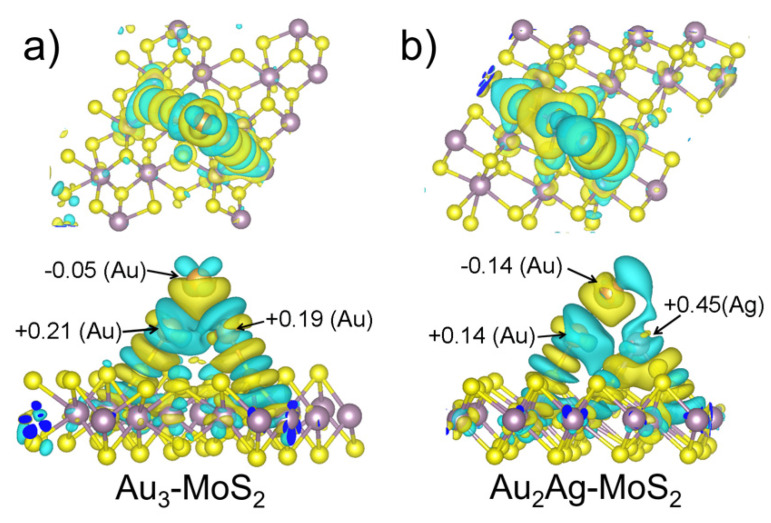
Top and side view of the isosurfaces of charge density differences of (**a**) Au_3_-MoS_2,_ and (**b**) Au_2_Ag-MoS_2_.

To explain the different catalytic efficiencies obtained we carried out periodic DFT calculations on model systems representing pristine MoS_2_ (metallic 1T phase), Au_2_Ag-MoS_2_ and Au_3_-MoS_2_, with the two latter models as simplified systems representing the bimetallic and monometallic nanoparticles grafted on the MoS_2_ surface (see Supplementary Materials, Figure S11). These calculations aim to correlate the changes of electronic density (Δρ) within noble metal nanoclusters and upon inclusion of the nanoclusters at the surface of MoS_2_, with the different catalytic activities. Figure 9 depicts the side and top views of the isosurfaces of charge density differences for models Au_2_Ag-MoS_2_ and Au_3_-MoS_2_. The charge depletion upon metal cluster bonding is localized at the cyan isosurfaces (positive charges), while the accumulation of negative charge is located at the yellow isosurfaces. If we analyze model Au_3_-MoS_2_ we observe a slight charge density transfer from the Au atoms bonded to sulfide towards the free Au atom (−0.05 net charge) and to the MoS_2_ surface. However, the analysis of model Au_2_Ag-MoS_2_ displays a different tendency, showing an important polarization of the charge density towards the naked Au atom in the cluster (−0.14 net charge) and, to a less extent, towards the S atoms of the semiconductor. In this case, an important loss of charge density is computed for the Ag atom (+0.45), whereas in contrast to the previous model, the Mo atoms also lose some charge density. Overall, a larger polarization of the charge density towards the naked gold sites is computed when a bimetallic Au_2_Ag cluster is computed instead of an Au_3_ monometallic one.

To summarize the quality of our results we have compared them to previously reported ones in Table 1, where catalyst composition, use of light, rate constant, amount of catalyst and activity factor (rate constant/amount of catalyst) are included.

As it can be observed, the catalytic performance (activity factor) of pristine MoS_2_ nanoflowers used in this work is ca. 7 times better than MoS_2_ nanosheets [23]. In contrast, porous MoS_2_ nanoparticles produce a larger catalytic activity, probably due to the higher surface/volume ratio and the increase of active sites of the porous structure [12]. Nevertheless, the inclusion in our case of ca. 2% atomic content of AuAg NPs produces a comparable effect with porous MoS_2_ NPs (activity factor 135.5 vs. 180 min^−1^·mg^−1^). On the other hand, when comparing with other metal-MoS_2_ hybrid nanostructures, the ones reported in this work, especially AuAg-MoS_2_ (**2**) nanohybrid, display better performance than those composed by Ag, Au, Pt or Pd and MoS_2_ NSs [23]. In the presence of NIR laser irradiation, a four-fold increase of activity factor is observed for porous MoS_2_ NPs and more than twice for the nanohybrids reported here using a slightly lesser irradiation power (3 vs. 2.5 W·cm^−2^).

It is important to note that the reduction of 4-NP into 4-AP is thermodynamically feasible and spontaneous since the reduction potentials for 4-NP/4-AP (0.76 V) and H_3_BO_3_/BH_4_^−^ (−1.33 V) versus normal hydrogen electrode make the process exergonic, where the change of the Gibbs free energy is negative (there is a release of free energy) [45]. However, this reaction is kinetically blocked without the concurrence of a catalyst due to high activation energy barriers, while the use of small amounts of small-size noble metal NP catalysts accelerates this transformation. Since this is a kinetically controlled process, the increase of the temperature through a photothermal effect would be directly related to higher rate constant values and faster reduction rates. The role of gold and gold-silver nanoparticles can be explained using DFT-periodic calculations (vide supra) that show an increase in charge density of a gold atom when an Au_3_ cluster interacts with a MoS_2_-1T surface, which is even more pronounced when an Au_2_Ag cluster is used. This charge density increases on gold, especially for the bimetallic nanoparticles, would explain the better ability of the nanohybrids to produce the electron transfer to 4-nitrophenolate, leading to a better reduction performance.

Given the results obtained in the catalytic reduction of 4-AP, when nanohybrids AuAg-MoS_2_ (**2**) and Au-MoS_2_ (**4**) are used as catalysts and they are compared to the pristine MoS_2_ NFs, a possible mechanism of photothermal-assisted catalytic activity of these materials can be proposed. First, PEG-stabilized MoS_2_ NFs material constitutes a good catalyst for this transformation. Indeed, as it has been previously reported for MoS_2_ nanosheets [23] the nitrophenolate ions are adsorbed at the surface of MoS_2_ through ion-dipole interactions by unsaturated S-atoms and, at the same time, the electrons and hydrogen atoms needed for the reduction of 4-NP are provided by the adsorbed BH_4_^-^ ions on the edge sites of MoS_2_ NFs. Then, H atoms can diffuse along the MoS_2_ surface reacting with the 4-NP molecules and transforming them into 4-AP ones. As it can be derived from previous results, the 1T phase of MoS_2_ [46,47] or, at least, a 1T/2H phase mixture is observed for these catalysts. In addition, the catalytic performance is strongly enhanced when AuAg and Au NPs are grafted to the surface of MoS_2_ NFs, especially in the case of alloyed AuAg NPs. The reason behind this behaviour would be related to the very good catalytic reduction ability of the bimetallic AuAg and Au NPs in the reduction of 4-NP [19] together with the reducing ability contribution of the MoS_2_ NFs. In addition, it is also likely that the heterojunction formed between MoS_2,_ and the metal nanoparticles would lead to a synergistic effect, favouring the electron transfer between MoS_2_ and the metal atoms in the NPs. In this way, more electron-rich Au atoms in the clusters (see DFT results above) would enhance their reducing ability towards 4-NP, whereas electron-deficient sites such as Ag or Mo atoms would favour the charge transfer between BH_4_^-^ ions and the catalyst. In addition, as we have commented in the Introduction section, the fact that AuAg NPs grafted on MoS_2_ NFs (**2**) produce a faster reduction rate than the corresponding Au ones (**4**) could be attributed to an electron enrichment in the gold near the interface by neighbouring silver atoms, in the absence of other factors like size, composition or surrounding environment. This suggestion agrees with our preliminary DFT results that show a larger electron enrichment of Au catalytic sites when bimetallic clusters are compared to monometallic ones. In the present study, the AuAg NPs display size of 2,1 ± 0.3 nm and a 2.0% atomic metal composition (1.3% atomic Ag and 0.7% atomic Au composition) in the nanohybrid **2** formed with PEG-stabilized MoS_2_ NFs, whereas Au NPs display size of 2.1 ± 0.9 nm and a 2.1% atomic metal composition in the same conditions in nanohybrid **4**, what rules out other factors enhancing the catalytic performance than the alloy composition.

In addition to the increased catalytic ability of the AuAg-MoS_2_ (**2**) and Au-MoS_2_ (**4**) nanohybrids compared to the pristine MoS_2_ NFs, a factor that greatly enhances the catalytic reduction rate of 4-NP for all the tested catalysts is the very efficient light-to-thermal energy conversion produced when MoS_2_ NFs and nanohybrids **2** and **4** are irradiated with NIR laser source (808 nm, 2.5 W·cm^−2^). This local temperature increase greatly accelerates the reduction rates of 4-NP for all tested catalysts. Low energy transfer of photons to electrons in the material produces this light-to-thermal energy conversion in MoS_2_ NFs. When this absorbed energy by the electrons does not produce electron release, an increase in the energy state of the electron or generation of another photon can be released as heat [48]. This process is very efficient in low band gap semiconductors such as MoS_2_ and it is even more favored when a fraction of the material crystallizes in the 1T metallic phase [49,50]. Therefore, when all the factors converge in catalyst AuAg-MoS_2_ (**2**) we found a very high value for the rate constant of reduction of 4-NP of 0.276 min^−1^, which can be attributed to the above-mentioned three factors: (i) synergistic effect of MoS_2_ and metal NPs in the catalysis; (ii) the use of bimetallic NPs instead of monometallic ones; and (iii) the high local photothermal heating of MoS_2_ NFs upon NIR laser irradiation.

## 4. Conclusions

We have shown that the combination of a hydrothermal method for the synthesis of MoS_2_ NFs with an organometallic route for the grafting of this nanomaterial with bimetallic AuAg or monometallic Au NPs constitutes a very robust approach for the preparation of multifunctional nanostructures oriented to the design of photothermal-assisted catalysts. Within this design, we can account for three factors that positively enhance the reduction rate of 4-nitrophenol into 4-aminophenol, which are: a synergistic effect of MoS_2_ and AuAg or Au NPs in the catalytic process; the preference for alloy AuAg NPs due to an increase of electron density on gold active sites; and the very efficient light-to-thermal energy conversion of MoS_2_ NFs that produces an important acceleration of the reduction rate.

## Figures and Tables

**Table 1:** Main characteristics and catalyst performance in the reduction of 4-NP of previously reported MoS_2_-based material and the ones reported in this work.

Sample	MoS_2_ Phase	Laser: λ (nm), Irrad (W·cm^−2^)	k × 10^2^ (min^−1^)	mg of Catalyst	Activity Factor k × 10^2^/Amount Cat. (min^−1^/mg)	Ref.
MoS_2_ NS	1T/2H	-	15.5	1.5	10.3	[23]
Ag-MoS_2_ NS	1T/2H	-	29.9	1.5	19.3	[23]
Au-MoS_2_ NS	1T/2H	-	18.8	1.5	12.5	[23]
Pt-MoS_2_ NS	1T/2H	-	19.6	1.5	13.1	[23]
Pd-MoS_2_ NS	1T/2H	-	38.6	1.5	25.7	[23]
ce-MoS_2_	main 1T	-	120	Not av.	Not available	[45]
Co-MoS_2_ NF	main 1T	-	90.8	0.25	363.2	[46]
porous MoS_2_ NP	2H	-	18.0	0.1	180.0	[12]
porous MoS_2_ NP	2H	808, 3.0	72.0	0.1	720.0	[12]
MoS_2_ NF	1T/2H	-	6.6	0.09	73.3	This work
AuAg-MoS_2_ NF	1T/2H	-	12.2	0.09	135.5	This work
Au-MoS_2_ NF	1T/2H	-	9.0	0.09	100.0	This work
MoS_2_ NF	1T/2H	808, 2.5	17.7	0.09	196.7	This work
AuAg-MoS_2_ NF	1T/2H	808, 2.5	27.6	0.09	306.7	This work
Au-MoS_2_ NF	1T/2H	808, 2.5	20.8	0.09	231.1	This work

NS = Nanosheets; ce = chemically exfoliated; NP = nanoparticles; NF = nanoflowers; 1T = metallic phase of MoS_2_; 2H = semiconducting phase of MoS_2_.

## Data Availability

Not applicable.

## Supplementary Materials

The following supporting information can be downloaded at: https://www.mdpi.com/article/10.3390/nano13061074/s1, Figure S1: Size histograms for AuAg NPs grafted on MoS_2_ NFs in nanohybrids **1**, **2**; Figure S2. HAADF-STEM images of AuAg-MoS_2_ nanohybrid **1**; Figure S3. TEM images and size histograms of Au NPs grafted on MoS_2_ NFs in nanohybrids **3** and **4**; Figure S4. Survey XPS spectrum for MoS_2_ NFs; Figure S5. Survey XPS spectrum for nanohybrid AuAg-MoS_2_ nanohybrid **1**; Figure S5. Survey XPS spectrum for nanohybrid AuAg-MoS_2_ nanohybrid **1**; Figure S6. Survey XPS spectrum for nanohybrid Au-MoS_2_ nanohybrid **3**; Figure S7. Narrow XPS spectra for Mo 3d, S 2p for MoS_2_ nanoflowers; Table S1. % atomic composition based on the XPS data for MoS_2_ NFs and nanohybrids AuAg-MoS_2_ **1** and Au-MoS_2_ **3**; Figure S8. Raman spectra for MoS_2_ NFs and nanohybrids **1**–**4**. (left) and table with assignment of the observed vibrational modes (right); Figure S9. On-off NIR laser irradiation cycles for nanohybrids **3** and **4**; Figure S10. Linear fit representations of -ln(A_t_/A_0_) vs. time at different temperatures; Figure S11. Top and side views of periodic DFT computed model systems (a) Au_3_-MoS_2_ and (b) Au_2_Ag-MoS_2_. (colour code: Mo green, S yellow, Au orange, Ag grey).
