# Supplementary material for: Boosting the Catalytic Performance of AuAg Alloyed Nanoparticles Grafted on MoS2 Nanoflowers through NIR-Induced Light-to-Thermal Energy Conversion
Source: Nanomaterials (Basel). 2023 Mar 16;13(6):1074. doi: 10.3390/nano13061074 (PMC10058585; doi:10.3390/nano13061074)
Supplement: Supplementary file 1 [file nanomaterials-13-01074-s001.zip › nanomaterials-2265125-supplementary.pdf]

Boosting the Catalytic Performance of AuAg Alloyed Nanoparticles Grafted on MoS_2 Nanoflowers through NIR-Induced Light-to-Thermal Energy Conversion

Sara Rodríguez-da-Silva[1], Abdel Ghafour El-Hachimi[1], José M. López-de-Luzuriaga[1], María Rodríguez-Castillo[1,*], Miguel Monge[1,*]

[1] Department of Chemistry. Centro de Investigación en Síntesis Químicas (CISQ). University of La Rioja, C/Madre de Dios 53, E-26006 Logroño, La Rioja, Spain.

* Correspondence: maria.rodriguez@unirioja.es (M.R.-C.);, miguel.monge@unirioja.es (M.M.)

SUPPLEMENTARY MATERIALS

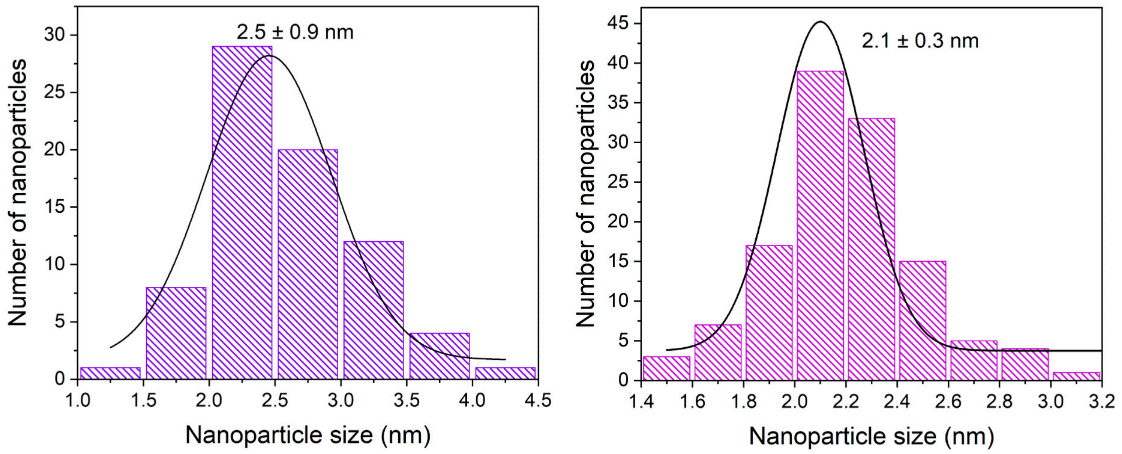

Figure S1. Size histograms for AuAg NPs grafted on MoS_2 NFs in nanohybrids **1**, **2**.

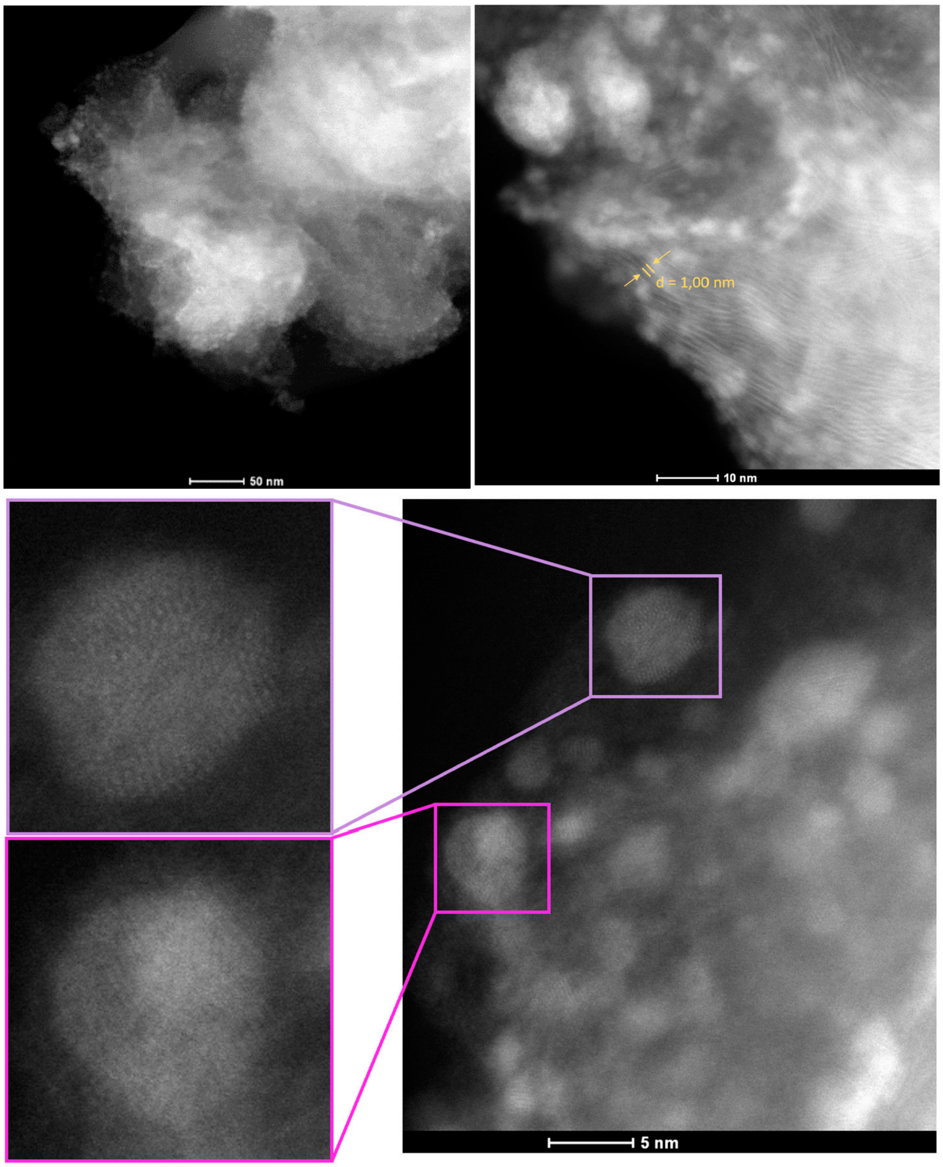

Figure S2. HAADF-STEM images of AuAg-MoS_2 nanohybrid **1**.

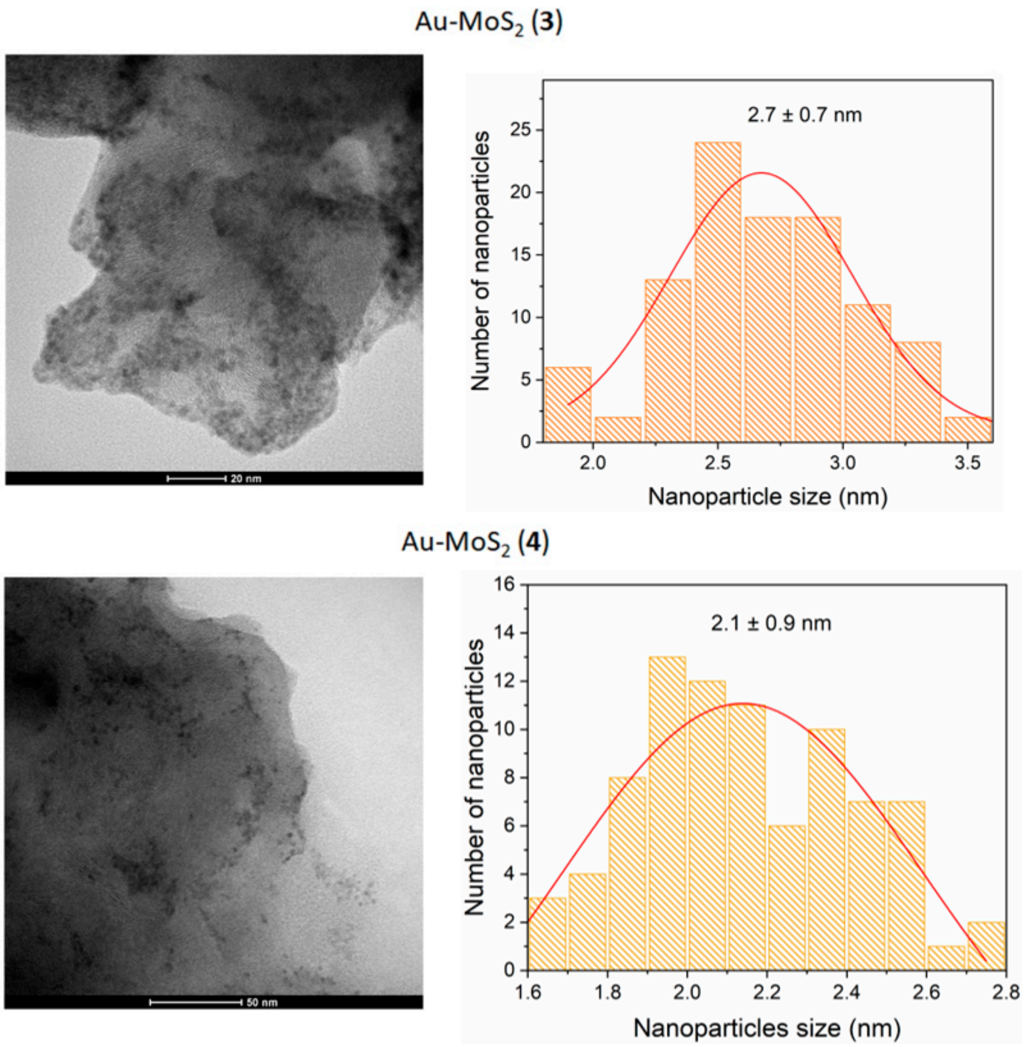

Figure S3. TEM images and size histograms of Au NPs grafted on MoS_2 NFs in nanohybrids **3** and **4**.

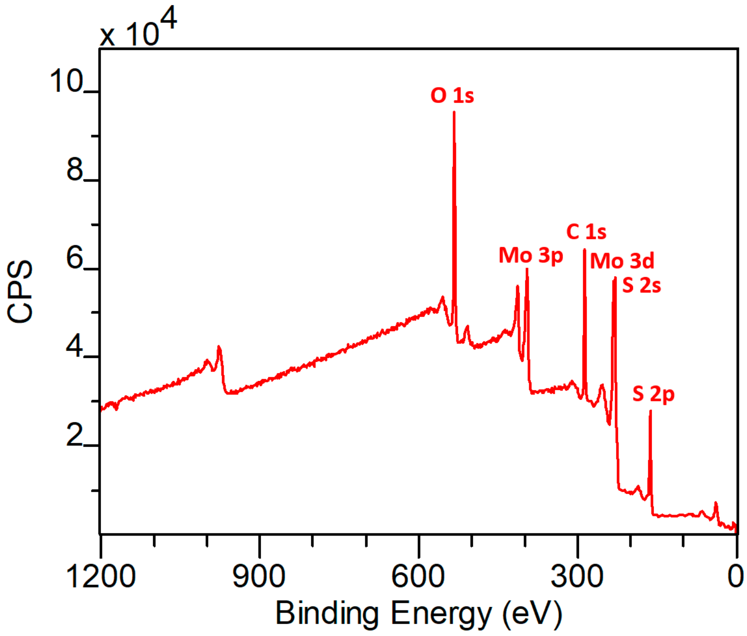

Figure S4. Survey XPS spectrum for MoS_2 NFs.

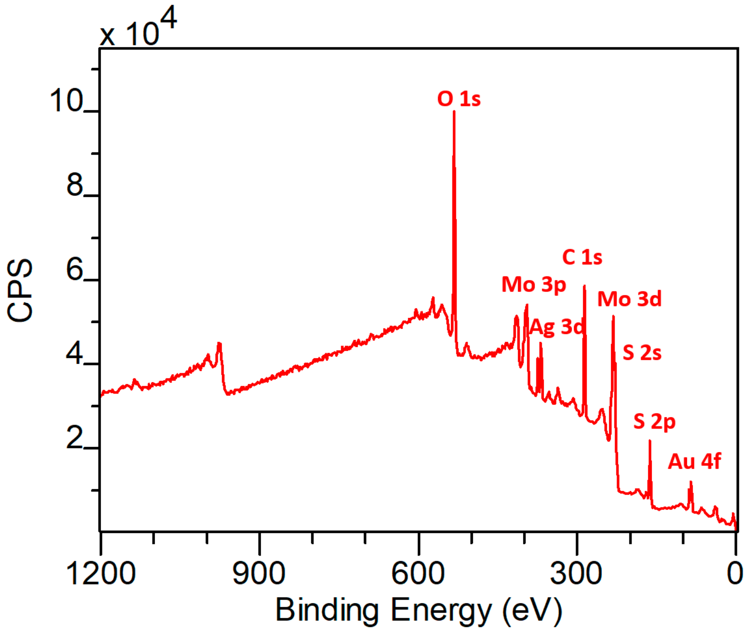

Figure S5. Survey XPS spectrum for nanohybrid AuAg-MoS_2 nanohybrid **1**.

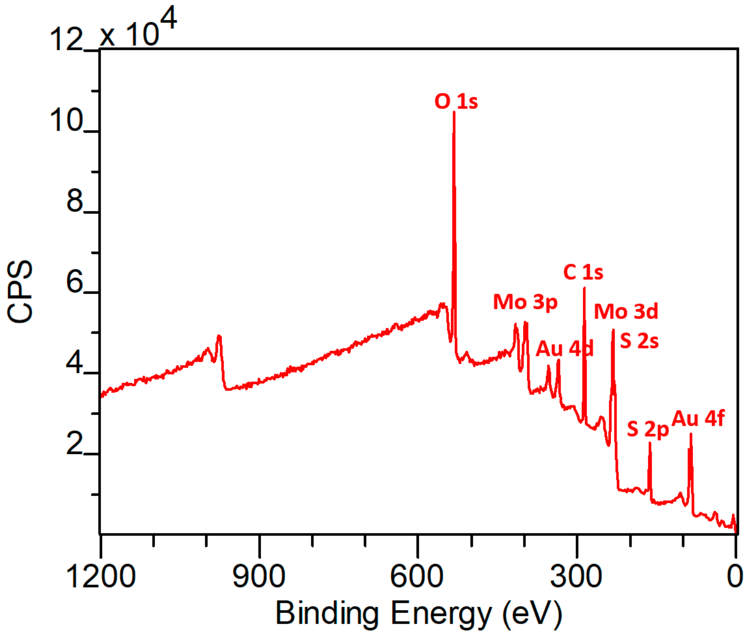

Figure S6. Survey XPS spectrum for nanohybrid Au-MoS_2 nanohybrid **3**.

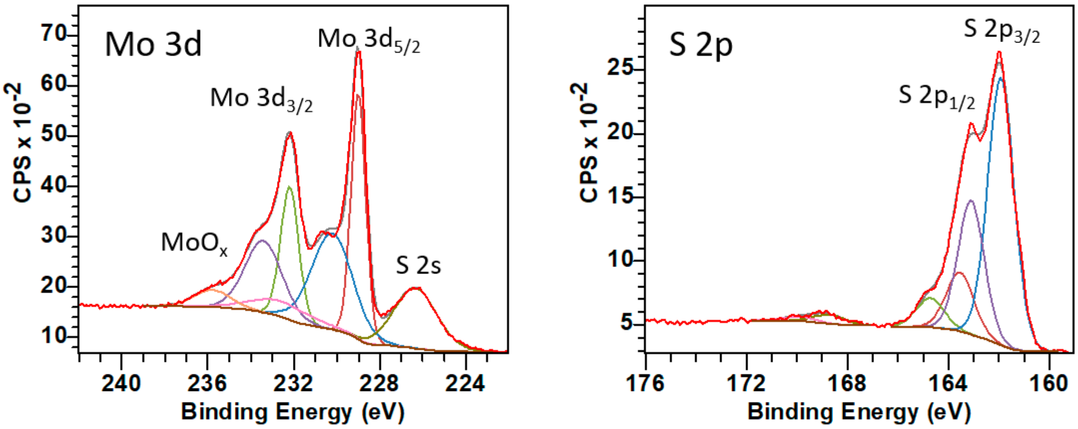

Figure S7. Narrow XPS spectra for Mo 3d, S 2p for MoS_2 nanoflowers.

Table S1. % atomic composition based on the XPS data for MoS_2 NFs and nanohybrids AuAg-MoS_2 **1** and Au-MoS_2 **3**.

Sample	C (at %)	O (at %)	Mo (at %)	S (at %)	Au (at %)	Ag (at %)
MoS_2 NFs	44.24	28.49	11.48	15.79	-	-
AuAg-MoS_2 **1**	44.33	31.72	9.55	11.78	0.75	1.23
Au-MoS_2 **3**	44.05	34.51	8.90	10.46	2.07	-

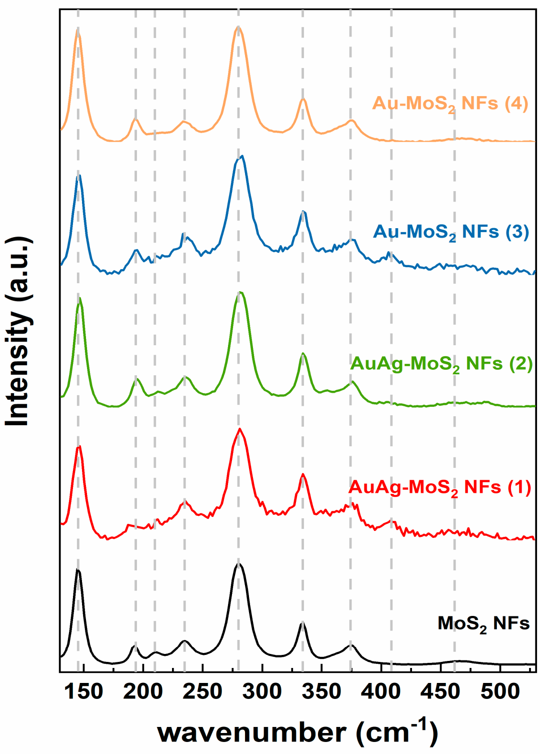

MoS_2	1	2	4	4	Mode
145	147	147	147	145	Phonon 1T
193	189	195	195	193	Phonon 1T
210	210	212	210	210	A_{1g} (1T)
235	235	235	233	233	LA (1T)
279	281	281	283	279	E_{1g} (1T)
334	334	334	334	334	Phonon 1T
374	374	376	374	374	E^1_{2g} (2H)
402	408	406	406	406	A_{1g} (1T, 2H)
460	462	460	462	464	2x LA (2H)

LA: Longitudinal acoustic phonon mode

Figure S8. Raman spectra for MoS_2 NFs and nanohybrids **1**-**4**.(left) and table with assignment of the observed vibrational modes (right).

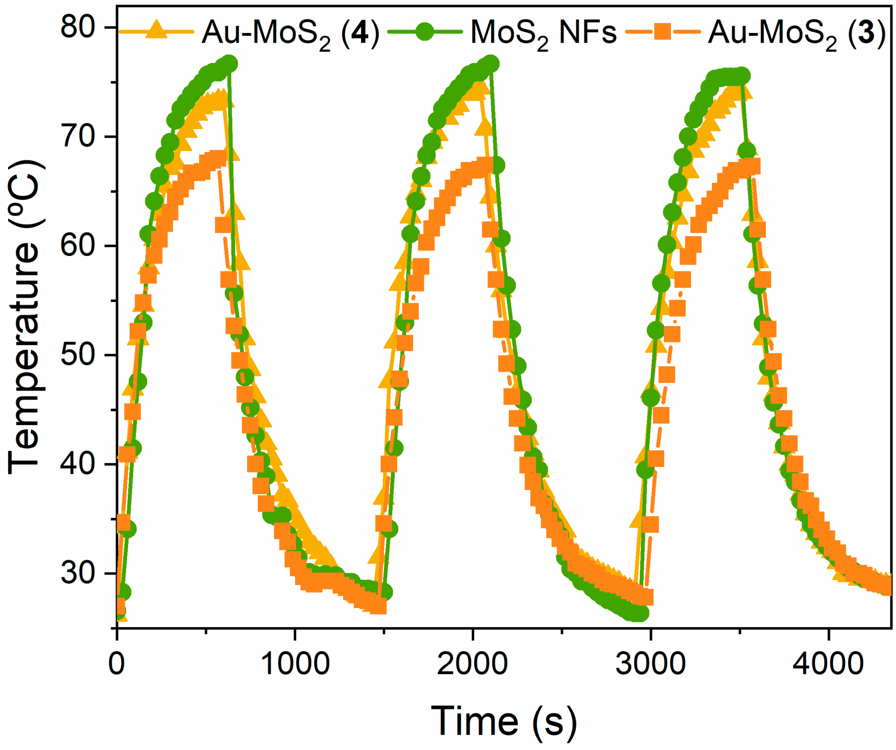

Figure S9. On-off NIR laser irradiation cycles for nanohybrids **3** and **4**.

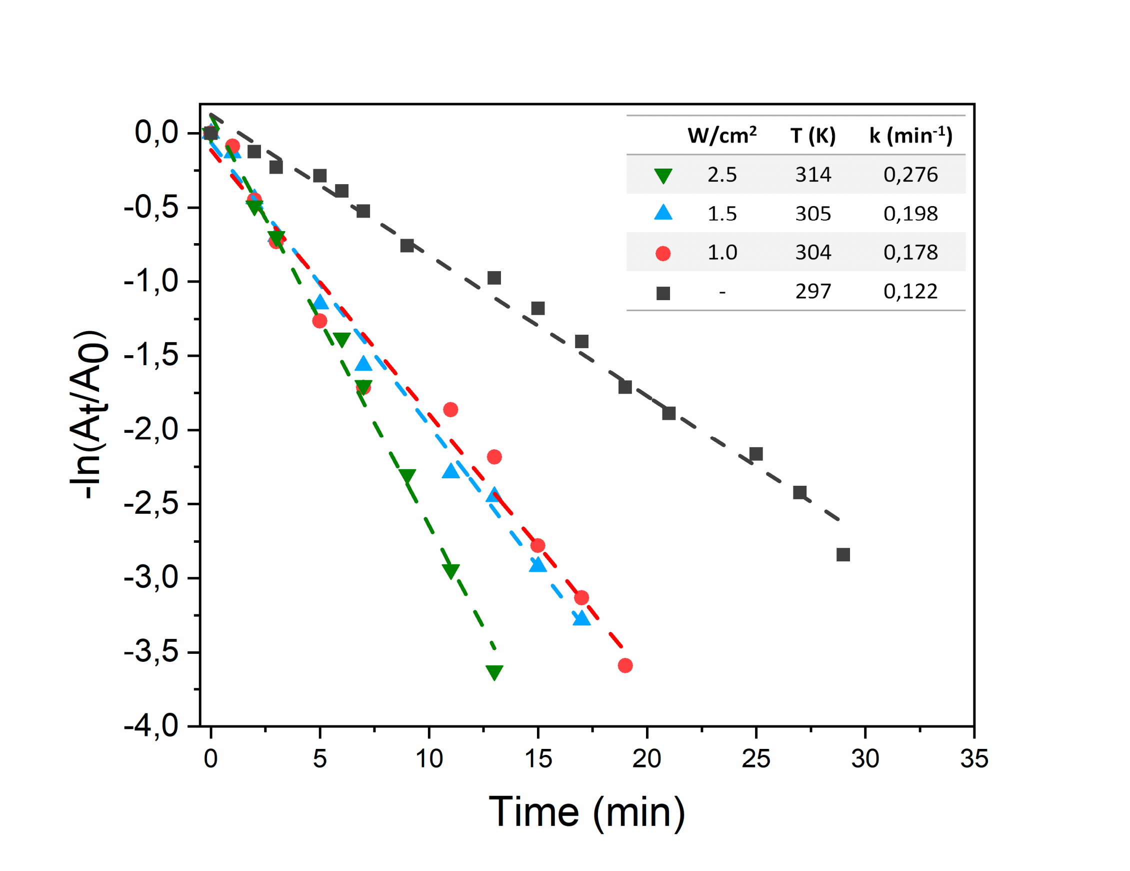

Figure S10. Linear fit representations of -ln(A_t/A_0) vs time at different temperatures.

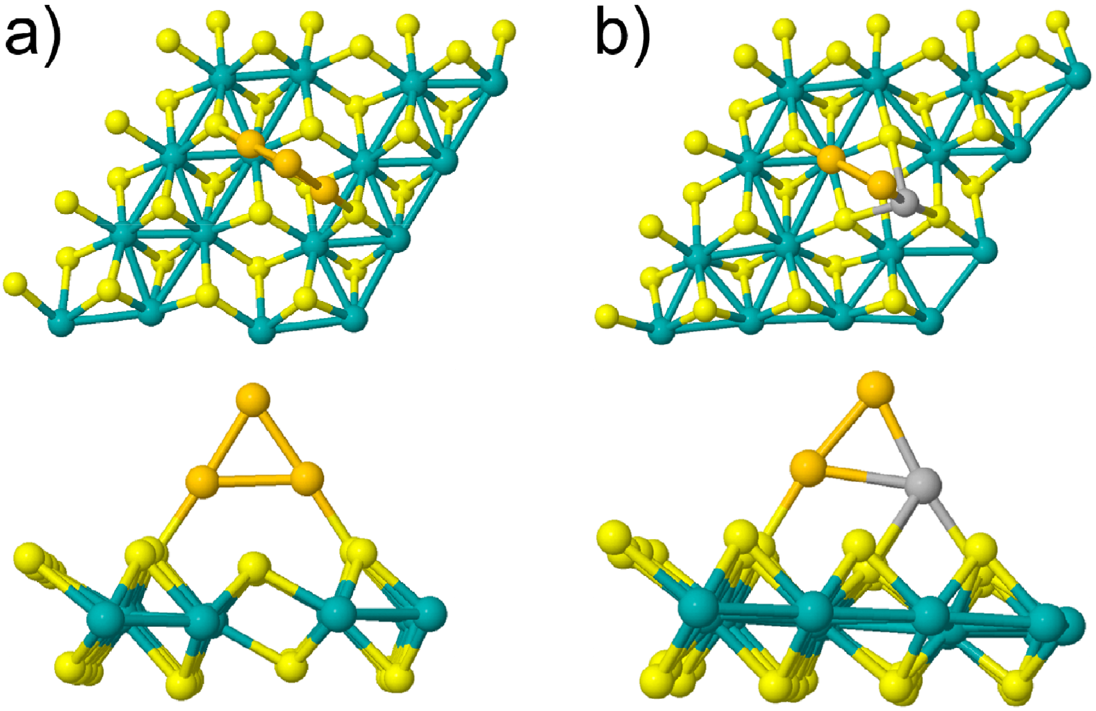

Figure S11. Top and side views of periodic DFT computed model systems (a) Au_3-MoS_2 and (b) Au_2Ag-MoS_2. (colour code: Mo green, S yellow, Au orange, Ag grey).
